# Uric acid‐induced pancreatic β-cell dysfunction

**DOI:** 10.1186/s12902-021-00698-6

**Published:** 2021-02-16

**Authors:** Asghar Ghasemi

**Affiliations:** Endocrine Physiology Research Center, Research Institute for Endocrine Sciences, Shahid Beheshti University of Medical Sciences, P.O. Box: 19395-4763, No. 24, Parvaneh Street, Velenjak, Tehran, Iran

**Keywords:** Uric acid, Pancreatic β-cell, Type 2 diabetes, Nitric oxide

## Abstract

Hyperuricemia is associated with insulin resistance, pancreatic β-cell dysfunction and consequently with development of type 2 diabetes. Although a direct relationship between high levels of uric acid (UA) and the development of diabetes is still a controversial issue, there is some evidence that strongly points to pancreatic β-cells damage as a result of high serum UA levels. Here, the mechanisms underlying UA-induced β-cell damage are discussed. Available literature indicates that UA can decrease glucose-stimulated insulin secretion and cause β-cell death. The mechanisms underlying these effects are UA-induced oxidative stress and inflammation within the β-cells. UA also stimulates inducible nitric oxide (NO) synthase (iNOS) gene expression leading to NO-induced β-cell dysfunction. Thus hyperuricemia may potentially cause β-cell dysfunction, leading to diabetes. It may be hypothesized that in hyperuricemic subjects, UA-lowering drugs may be beneficial in preventing diabetes.

## Background

Worldwide, the prevalence of diabetes is about 8–9 % [1, 2] and its incidence varies between 2.9 and 23.5 per 1000 population [3]. The worldwide prevalence of gout, defined as deposition of monosodium urate crystals mostly in the peripheral joints, ranges from 0.1 to 10 %, and its incidence varies 0.3 to 6 per 1000 person-years [4]. Both prevalence [5] and incidence [6] of diabetes are higher in patients with gout.

Uric acid (UA) is the end product of exogenous and endogenous purine (adenine and guanine) metabolism [7, 8]. The liver and the intestine are the major sites of endogenous UA production [9], which is about 300–400 mg/day [8]. Dietary contribution is approximately 300 mg/day with a total pool size of 1200 mg in men and 600 mg in women [10, 11]. UA homeostasis depends on a balance between production and catabolism [7], where 20–40 % of UA is excreted by the gastrointestinal tract and 60–80 % by the kidneys [7, 12]. Secreted UA by the intestine is further metabolized by the gut bacteria (intestinal uricolysis) [12]. UA is freely filtrated by the kidneys, of the filtrated load (plasma concentration of UA × glomerular filtration rate), 90 % is reabsorbed and therefore, fractional excretion of UA is about 10 % (7–12 %) [8, 12, 13]. Physiological functions of UA include but not limited to antioxidant property [9, 14], defense against neurological diseases [14], autoimmune diseases [9], and maintaining endothelial function [9].

High serum UA levels is a risk factor for type 2 diabetes mellitus (T2DM) as reported in different population-based studies [15–18]. According to meta-analyses of cohort studies, each 1 mg/dL (59.48 µmol/L) increase in serum levels of UA increases the risk of developing T2DM by about 6–17 % [19–21]. High UA concentrations is associated with both insulin resistance [16, 22] and β-cell dysfunction [23], two defects that are at the core of pathophysiology of T2DM [24]. In healthy subjects with normal serum UA concentrations, a positive correlation between serum UA levels and steady-state plasma glucose (SSPG) concentrations, an index of insulin resistance, has been reported [25]. In addition, renal clearance of UA is inversely associated with insulin resistance [25]. A direct relationship between changes in UA homeostasis and diabetes is still controversial [26, 27]. Using a multilocus Mendelian randomization approach, it has been shown that for each 1 mg/dL increase in circulating UA concentrations, there is an associated 20 % higher risk of diabetes, but the data does not support causality [28]. However, this approach to show potential causality has been criticized as it may dissociate the physiological serum-intracellular relationship [26]. In addition, acute euglycemic hyperinsulinemia decreased fractional UA excretion by 26 % (from 6.1 ± 0.8 % to 4.5 ± 0.6 %) in healthy subjects, indicating that insulin inhibits renal UA excretion [29] and that high UA levels causes insulin resistance by affecting the insulin signaling pathways [22]. Although a cause or effect relationship between hyperuricemia and diabetes is still a matter of debate, some experimental evidence indicates that high UA levels can damage pancreatic β-cells; this review aims to summarize the mechanisms underlying UA-induced β-cell damage.

### Uptake of uric acid by pancreatic β-cells

Urate transporters include, (i) urate transporter 1/solute carrier family 22, member 12 (URAT1/SLC22A12), (ii) ATP-binding cassette subfamily G, member 2/breast cancer resistance protein (ABCG2/BCRP), and (iii) glucose transporter 9 (GLUT9/SLC2A9) [8]. Expression of URAT1 in endocrine pancreas is controversial; both low expression in pancreatic islets of rat [30] or no expressions in pancreatic β-cell lines (INS-1 cells and RIN-m5F cells) [31] have been reported. On the other hand, both variants of GLUT9 (GLUT9a and GLUT9b) are expressed in mouse insulinoma MIN6 cells, mouse islets, and human islets [32]. In addition, GLUT9 expression in pancreatic β-cells is specific [32]. Although human GLUT9 is a urate transporter [7], this carrier also participates in pancreatic β-cells function, as its knockdown resulted in reduced cellular ATP levels that correlated well with reductions in glucose-stimulated insulin secretion (GSIS) in MIN6 and INS cells [32].

### Uric acid and β-cell dysfunction

In 1948, Griffiths reported that feeding rabbits with a diet that was deficient in methionine and cystin for 6–7 weeks decreased blood glutathione levels by about 40–53 % [33]. Intraperitoneal injection of UA (1 g/kg) to these rabbits increased blood glucose concentrations to hyperglycemic levels, and therefore, it has been suggested that UA exerts a diabetogenic action [33]. It has also been shown that inhibition of uricase (urate oxidase) in rats, along with UA feeding, increased serum glucose and decreased serum insulin, and therefore, decreased insulin:glucose ratio [34]. Uricase-knockout mice have glucose intolerance and are more susceptible to development of diabetes [23, 35]. In addition, in hyperuricemic subjects, β-cells fail to compensate variations of insulin sensitivity [36].

### Inhibitory effect of uric acid on glucose‐stimulated insulin secretion

UA inhibits GSIS in isolated pancreatic rat islets [37, 38], pancreatic mouse islets [39], and pancreatic β-cell lines including Min6 cells [39, 40] and INS-1 cells [31, 38]. Inhibition varies between 30 and 80 % depending on the dose of UA, time of exposure, and different cell lines or different animal studied. High UA concentrations decreases GSIS by about 30–42 % in Min6 cells [39, 40], 44 % in isolated mouse islets [39], and 80 % in isolated rat islets [37]. Decreased GSIS in hyperuricemia may be due to decreases in MafA protein expression [39] as MafA is a key regulator of insulin secretion in β-cells [41].

The association between UA and insulin secretion is quite complex. It has been shown that UA increases GSIS in isolated perfused rat pancreas [42]. In addition, a positive correlation between serum UA and total insulin secretion has been reported using a hyperglycemic clamp technique in type 2 diabetic patients without hyperuricemia [43]. The effects of high UA levels on basal insulin secretion are not consistent. Inhibition in rat pancreatic islets [34, 37] and INS-1 cells [31] as well as no effects in INS-1 cells [38], Min6 cells [40], and isolated rat islets [38], have been reported.

### Uric acid and β-cell death

**Fig. 1:**
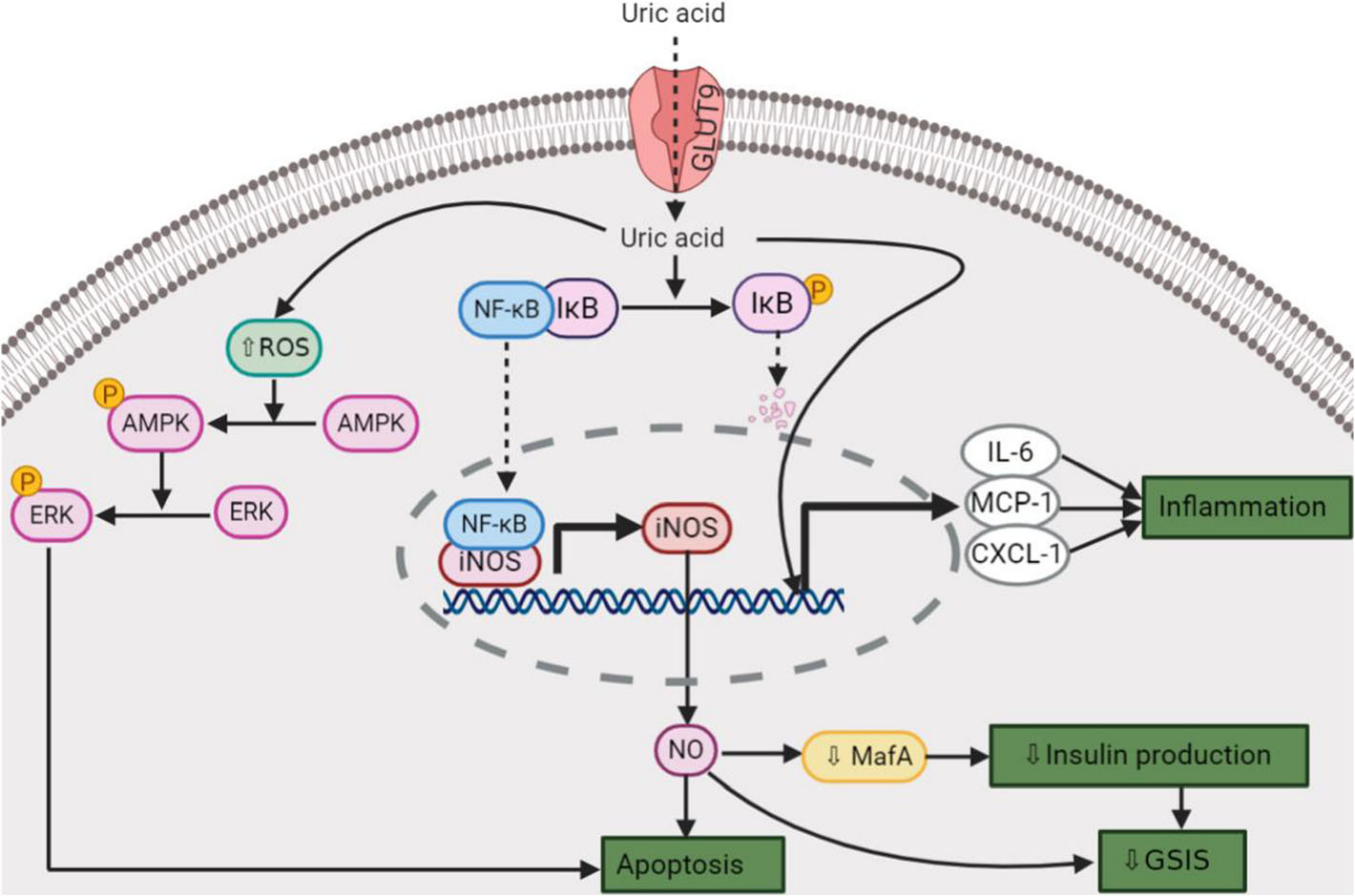
Mechanisms underlying uric acid (UA)-induced β-cell dysfunction. UA probably enters the β-cells via glucose transporter 9 (GLUT9). Intracellular UA increases reactive oxygen species (ROS), which phosphorylates and activates AMP-activated protein kinase (AMPK) and then extracellular signal-regulated kinase (ERK). Phosphorylated ERK causes β-cell apoptosis. UA also phosphorylates and degrades inhibitor of kappa B (IκB) that permits the transcription factor nuclear factor kappa B (NF-κB) to enter the nucleus and increases expression of inducible nitric oxide synthase (iNOS). NO overproduction decreases glucose-stimulated insulin secretion (GSIS) and causes β-cell apoptosis. CXCL-1, chemokine (C-X-C motif) ligand 1; MCP-1, monocyte chemoattractant protein-1; IL-6, interleukin-6. *Created with BioRender.com*

In addition to decreased GSIS, other mechanisms are involved in hyperuricemia-induced β-cell dysfunction, development of glucose intolerance, and T2DM. These include, increased inducible nitric oxide (NO) synthase (iNOS)-derived NO production [39, 40], increased inflammation [30, 39], increased oxidative stress [30, 31, 38], and increased apoptosis and β-cell death [39, 40]. These underlying mechanisms can be categorized under two major pathways that are activated by UA (Fig. 1): (1) The nuclear factor kappa B (NF-κB)-iNOS-NO signaling pathway, and (2) Reactive oxygen species (ROS)-AMP-activated protein kinase (AMPK)-extracellular signal-regulated kinase (ERK) signaling pathway.

In Min6 cells, UA activates the NF-κB signaling pathway by phosphorylation and degradation of inhibitor of κB (IκB) [39]; NF-κB increases iNOS expression and therefore NO production, which causes a decrease in GSIS and β-cell apoptosis [39]. In RIN-m5F cells, UA increases the mRNA expression of inflammatory mediators, including chemokine (C-X-C motif) ligand 1 (CXCL-1 or KC), monocyte chemoattractant protein-1 (MCP-1), and interleukin-6 (IL-6) [30].

High levels of UA inhibit the growth of the pancreatic β-cell lines (INS-1 and RIN-m5F) in a time- and dose-dependent manner via the ROS-AMPK-ERK signaling pathway [31]. High concentrations of UA also induce oxidative stress in these cell lines [31]. Elevated ROS increases phosphorylation of AMPK, which in turn increases ERK phosphorylation [31], thus inhibiting the cell growth [31]. Luteolin (a flavonoid), by suppressing UA-activated NF-κB-iNOS-NO signaling pathway [44], and resveratrol (a polyphenolic compound), by increasing miR-126 expression [40], protect the pancreatic β-cells from UA-induced dysfunction.

### Uric acid and nitric oxide

It has been shown that the timing of serum UA peak (5:08) and serum NO nadir (5:32) coincide in healthy men, suggesting that their concentrations are physiologically related [45]. In addition, in male rats, serum UA levels are inversely correlated with serum NO metabolites, with hyperuricemia decreasing serum NO metabolite levels by about 40–50 % [46]. More details regarding circadian variations of NO metabolites can be found elsewhere [47].

UA increases iNOS expression in the β-cells, decreases GSIS, and causes apoptosis [39]. However, the potential role of NO in UA-induced β-cell dysfunction needs further investigations. NO produced by different NOS isoforms (i.e. endothelial NOS, neural NOS, and iNOS) exerts different effects on β-cell function [48], and in most cases, the eNOS/nNOS-derived NO has physiological relevance, whereas iNOS-derived NO in general has pathological effects. In endothelial cells, high UA levels decreases NO production [46, 49, 50], increases arginase activity [49], and suppresses insulin-stimulated phosphorylation of PKB (Akt) and eNOS [51]. In addition, in human umbilical vein endothelial (HUVEC) cells, a high concentration of UA causes mitochondrial calcium overload probably by switching the direction of mitochondrial sodium-calcium exchanger (NCX_mito_) function from efflux to influx. This calcium overload increases ROS production, which decreases eNOS expression and NO release, causing endothelial dysfunction [52]. Because NCX_mito_ is involved in insulin secretion from β-cells [53], one can speculate that hyperuricemia can affect β-cell function via this pathway. However, further studies are needed to confirm these effects in the β-cells.

### Uric acid‐lowering drugs in diabetes

Considering UA as a target for prevention/management of diabetes is still premature and needs to be evaluated in clinical trials. However, several lines of evidence indicate a potential favorable outcome of these drugs in diabetes. Zurapamic, an inhibitor of UA reabsorption in the kidneys, protects INS-1 cells and rat islets against UA-induced damage by decreasing URAT1 expression and oxidative stress [38]. Allopurinol, a competitive inhibitor of xanthine oxidase that decreases UA production, protects isolated islets from neonatal rats against the cytotoxic effects of styreptozotocin, probably via decreasing intracellular UA levels [54]. Benzbromarone, an uricosuric drug, inhibits fatty acid-binding protein 4 and improves glucose tolerance in type 2 diabetic db/db mice [55]. Allopurinol improves endothelial function in hypertensive type 2 diabetic patients [56]. In a retrospective cohort, it has been shown that compared with non-users, incidence of new-onset diabetes is lower in patients with gout being treated with benzbromarone [6].

## Conclusions and perspectives

UA induces oxidative stress, the inflammatory response in the β-cells, and decreases GSIS, causing β-cell apoptosis. The threshold theory for the actions of UA on the β-cells hypothesizes that the detrimental effects of UA occurs above a given concentration. In support of this notion, it has been shown that the inhibitory effect of UA on GSIS in rat pancreatic islets has a sudden occurrence at a threshold of 6.7 mg/dL (0.4 mM) [37]. Other hypothesis of a potential association between UA and diabetes is that the effects of hyperuricemia, are potentiated in presence of other risk factors such as obesity or in genetically at risk subjects [34]. In support of this concept, a positive association has been found between serum UA levels and the body mass index [57]. Also, an association between serum UA levels and glucose homeostasis has been shown to be mediated by adiposity [58].

Regarding the association between UA and β-cell function, the effects of UA on the genes and proteins that are involved in insulin biosynthesis and secretion warrants further investigations. In addition, most mechanistic findings have been drawn from in vitro studies or from animal studies. As always, it should be noted that extending results from animal studies to humans needs abundance of caution, as UA metabolism is different between humans and rodents [59]. Unlike humans, rodents have uricase, and therefore, degrade UA more rapidly [59]. Thus, circulating UA concentrations in humans is about 5–20 fold higher than in most other mammals [12, 13].

All in all, hyperuricemia may potentially cause β-cell dysfunction and predispose subjects to metabolic disorders such as diabetes. If this holds true, then UA-lowering drugs may be helpful in prevention/management of diabetes, at least in subjects who are at risk for both hyperuricemia and diabetes.

## Data Availability

Not Applicable.

## List of Abbreviations

ABCG2: ATP-binding cassette subfamily G, member 2
AMPK: AMP-activated protein kinase
BCRP: Breast cancer resistance protein
CXCL-1: Chemokine (C-X-C motif) ligand 1
eNOS: Endothelial nitric oxide synthase
ERK: Extracellular signal-regulated kinase
GLUT9: Glucose transporter 9
GSIS: Glucose-stimulated insulin secretion
IL-6: Interleukin-6
MCP-1: Monocyte chemoattractant protein-1
NCX_mito_: mitochondrial sodium-calcium exchanger
NF-κB: Nuclear factor kappa B
nNOS: Neural nitric oxide synthase
NO: Nitric oxide
NOS: Nitric oxide synthase
ROS: Reactive oxygen species
SLC: Solute carrier family
SSPG: Steady-state plasma glucose
T2DM: Type 2 diabetes mellitus
URAT1: Urate transporter 1
